# Circadian rhythms in *Per1*, PER2 and Ca^2+^ of a solitary SCN neuron cultured on a microisland

**DOI:** 10.1038/s41598-019-54654-5

**Published:** 2019-12-04

**Authors:** Yoshihiro Hirata, Ryosuke Enoki, Kaori Kuribayashi-Shigetomi, Yoshiaki Oda, Sato Honma, Ken-ichi Honma

**Affiliations:** 1 Photonic Bioimaging Section, Hokkaido University Graduate School of Medicine, Sapporo, Japan; 2 Institute for the Advancement of Higher Education, Hokkaido University, Sapporo, Japan; 3 Graduate School of Information Science and Technology, Hokkaido University, Sapporo, Japan; 4 Department of Chronomedicine, Hokkaido University Graduate School of Medicine, Sapporo, Japan; 5 Research and Education Center for Brain Science, Hokkaido University, Sapporo, Japan; 6 Biophotonics Research Group, Exploratory Research Center on Life and Living Systems (ExCELLS), National Institutes of Natural Sciences, Okazaki, Japan; 7 Division of Biophotonics, National Institute for Physiological Sciences, National Institutes of Natural Sciences, Okazaki, Japan; 8 Present Address: Department of Oral Chrono-Physiology, Unit of Basic Medical Sciences, Graduate School of Biomedical Sciences, Nagasaki University, Nagasaki, Japan

**Keywords:** Circadian mechanisms, Circadian rhythms

## Abstract

Circadian rhythms in *Per1*, PER2 expression and intracellular Ca^2+^ were measured from a solitary SCN neuron or glial cell which was physically isolated from other cells. Dispersed cells were cultured on a platform of microisland (100–200 μm in diameter) in a culture dish. Significant circadian rhythms were detected in 57.1% for *Per1* and 70.0% for PER2 expression. When two neurons were located on the same island, the circadian rhythms showed desynchronization, indicating a lack of oscillatory coupling. Circadian rhythms were also detected in intracellular Ca^2+^ of solitary SCN neurons. The ratio of circadian positive neurons was significantly larger without co-habitant of glial cells (84.4%) than with it (25.0%). A relatively large fraction of SCN neurons generates the intrinsic circadian oscillation without neural or humoral networks. In addition, glial cells seem to interrupt the expression of the circadian rhythmicity of intracellular Ca^2+^ under these conditions.

## Introduction

The circadian rhythms in the suprachiasmatic nucleus (SCN), the central clock of the mammals, are considered to be based on single neurons with an intrinsic circadian oscillation, which are integrated by mutual synchronization through the neural or humoral mechanism^1,2^. However, it is still a matter of discussion whether (1) only a fraction of SCN neurons in the SCN is capable of generating intrinsic rhythms and the most of other cells are just enforced or driven by a set of oscillating neurons^3^, (2) the circadian rhythms in the oscillating neurons are noisy and unstable^3,4^, or (3) there is a class of SCN neurons with clearly separable circadian periods^5–7^. In addition, very little is known about the neural and humoral networks in the SCN, which couple the oscillating or non-oscillating neurons in neighbors, regions or the whole SCN. It is also not well understood the roles the oscillating glial cells in the neuronal circadian rhythm expression from the SCN^8–11^. To have a better understanding of the circadian organization in the SCN, we need to monitor the circadian rhythm in solitary SCN neuron which is physically (e.g., via synapses and gap junction) separated from other cells and to characterize the oscillating neurons. However, a very few circadian rhythms have been reported in a fully isolated single SCN neuron^3^, which was definitely attributable to technical difficulties.

A molecular auto-feedback loop (core loop) is widely accepted as an origin of circadian oscillation, in which clock genes, *Periods* (*Pers*), *Cryptochromes* (*Crys*), *Bmal1*, *Clock* and their protein products are involved^12,13^. The dynamics of the core loop could be influenced by many factors such as the accumulation and/or degradation of clock gene products^14^, the interlocked auto-feedback loops such as the *Bmal1-Rev* loop^15,16^, transcriptional elements^17,18^, and the inputs from the neural networks as well as the neighboring glial cells^19^. Any change in the loop dynamics may alter the circadian period, amplitude and other characteristics of circadian oscillation. For instance, the intracellular Ca^2+^ levels show a robust circadian rhythm^20–22^, which are located at the downstream of the core loop^20^. Further, the Ca^2+^ levels are changed in response to the input from neural networks^22–24^, which resets the circadian oscillation^24^. Thus, the circadian rhythms in intracellular Ca^2+^ play a gating role in circadian integration of the SCN neurons and are critically important to understand the interaction between the intrinsic cellular oscillation and external coupling or entraining inputs.

Functional synapses are crucial for mutual synchronization of oscillating neurons in the SCN^1^. Distribution of circadian periods is very narrow in individual mice or rats under constant conditions, but becomes wider in individual cells of the SCN slice culture and spread through a broad range in dispersed culture cells^4,25,26^. The density of cells in culture affects the ratio of circadian rhythm positive neurons, and the lower the density the smaller the rhythm positive neurons^3^. These results suggest that only a fraction of the SCN neurons is intrinsically oscillating neurons and the neural networks reinforce or drive the non-oscillating neurons to express circadian rhythms. Tetrodotoxin (TTX) blocks the sodium channel dependent neural firing and thereby shutdown the neural input from the networks^27^. TTX treatment to the SCN slice culture abolishes the circadian rhythms in almost half of the neurons by 7-day treatment^28^, reduces the amplitude of surviving rhythm and desynchronizes them^20^. The washout of TTX recovers the amplitude though not to the full range and resynchronizes the cellular rhythms in several days^28,29^. These findings also support the idea that not all SCN neurons are intrinsic circadian oscillators and neural networks integrate the circadian rhythms in the SCN. By contrast, the circadian oscillation *in vivo* persists even when TTX is applied to the SCN^30^ suggesting the integration of oscillating cells is much stronger *in vivo* than *ex vivo*.

The humoral factors such as arginine vasopressin (AVP) and vasoactive intestinal polypeptide (VIP) are also involved in the coupling of oscillating SCN neurons to express the coherent circadian rhythms^31,32^. Co-culture of an intact SCN slice with a genetically aperiodic SCN restores the circadian rhythm^33^ through AVP or VIP receptor mechanism^32^. However, the mechanism of humoral coupling is largely unknown as for the route, the limit distance of effectiveness and the necessary dose for coupling. Humoral factors are transported generally in the extracellular space depending on the concentration gradient and a diffusion coefficient.

The SCN glial cells also show robust circadian rhythms in clock gene expression, ATP and glutamate release^9,34,35^ but the role of glial circadian rhythms is still a matter of debate. Glial cell-specific knock-down of *Bmal1* gene expression does not affect the circadian oscillation in the SCN neurons but lengthens the circadian period^11^. Whether this is due to the uncoupling of oscillating neurons is not elucidated. Recently, the glial circadian rhythm is reported to enable functionless SCN neurons to oscillate and express behavioral rhythms^8^, indicating that the circadian rhythms of SCN glial cells are capable of interacting with the circadian oscillation of SCN neurons^19^.

In the present study, we introduced two different techniques for single cell culture to monitor the circadian rhythm in a solitary SCN neuron which was physically isolated from other cells, without any synaptic contact or gap junction. To this end, we are able to demonstrate for the first time the robust circadian rhythms in *Per1*, PER2 expression and intracellular Ca^2+^ in a large fraction of solitary SCN neurons. We also found that the glial cells interact with the SCN neurons to disorganize the circadian rhythm expression in intracellular Ca^2+^.

## Results

### Culture of a solitary SCN neuron and glial cell

We applied two different techniques of cell culture, a collagen spraying method and photolithographic microfabrication method, to monitor circadian rhythms from a single solitary cell (Supplementary Fig. 1). The difference was in the method of making small islands (microislands) in a culture dish, to which a single cell adhered.

### Circadian oscillation in a solitary SCN neuron

**Figure 1:**
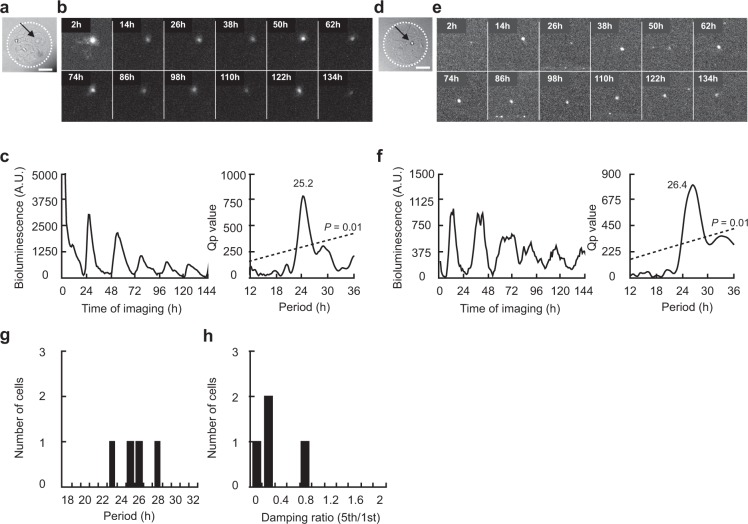
Circadian rhythm in *Per1-luc* expression in a solitary SCN neuron. (**a**) Bright-field photomicrograph of a solitary neuron on a microisland. The border of the microisland is indicated by a white dotted circle. Scale bar shows 100 µm. (**b**) Time-lapse images of *Per1-luc* bioluminescence at every 12 hr from the same neuron as in (**a**). (**c**) Circadian rhythm of *Per1* expression in the same neuron as in (**b**) (left) and Chi-square periodogram (right). (**d**) Bright-field photomicrograph of another solitary neuron. (**e**) Time-lapse images of bioluminescence from the same neuron as in (**d**). (**f**) Circadian rhythm in *Per1* expression in the same neuron as in (**e**) (left) and Chi-square periodogram (right). (**g**) Distribution of circadian period in solitary neurons (*n* = 4) and (**h**) of damping ratio.

A single neuron with the *Per1*-*luc* reporter (*Per1* neuron) was identified in 8 islands out of 11 examined, which was made by collagen spraying method. Among them, one neuron survived for only 93 hours and was not used for further analyses (see Methods). A significant circadian rhythm was detected in 4 solitary neurons (57.1%) by chi-square periodogram (Fig. 1 and Supplementary Fig. 2a). The circadian period was ranging from 23.6 h to 28.0 h. The mean period (±SD) was 25.8 ± 1.8 h (Fig. 1g). The variability of cycle intervals in terms of SD was 2.3 ± 1.1 h and the damping ratio in terms of the ratio of amplitudes on the first and the fifth cycle was 0.4 ± 0.3. Two islands contained two neurons, where the circadian rhythm was positive in both neurons in one island and negative in both in the other. One island contained three neurons, one of them was circadian rhythm negative but other two were untractable.

**Figure 2:**
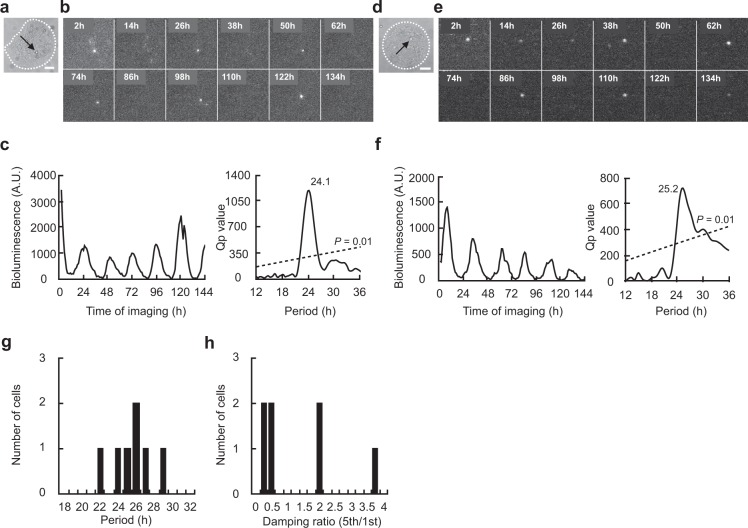
Circadian rhythm in PER2::LUC expression in a solitary SCN neuron. (**a**) Bright-field photomicrograph of a solitary neuron on a microisland. The border of the microisland is indicated by a white dotted circle. Scale bar shows 100 µm. (**b**) Time-lapse images of PER2::LUC bioluminescence at every 12 hr from the same neuron as in (**a**). (**c**) Circadian rhythms of PER2::LUC expression in the same neuron as in (**b**) (left) and Chi-square periodogram (right). (**d**) Bright-field photomicrograph of another solitary neuron. (**e**) Time-lapse images of bioluminescence from the same neuron as in (**d**). (**f**) Circadian rhythm in PER2::LUC expression in the same neuron as in (**e**) (left) and Chi-square periodogram (right). (**g**) Distribution of circadian period in solitary neurons (*n* = 7) and (**h**) of damping ratio.

**Figure 3:**
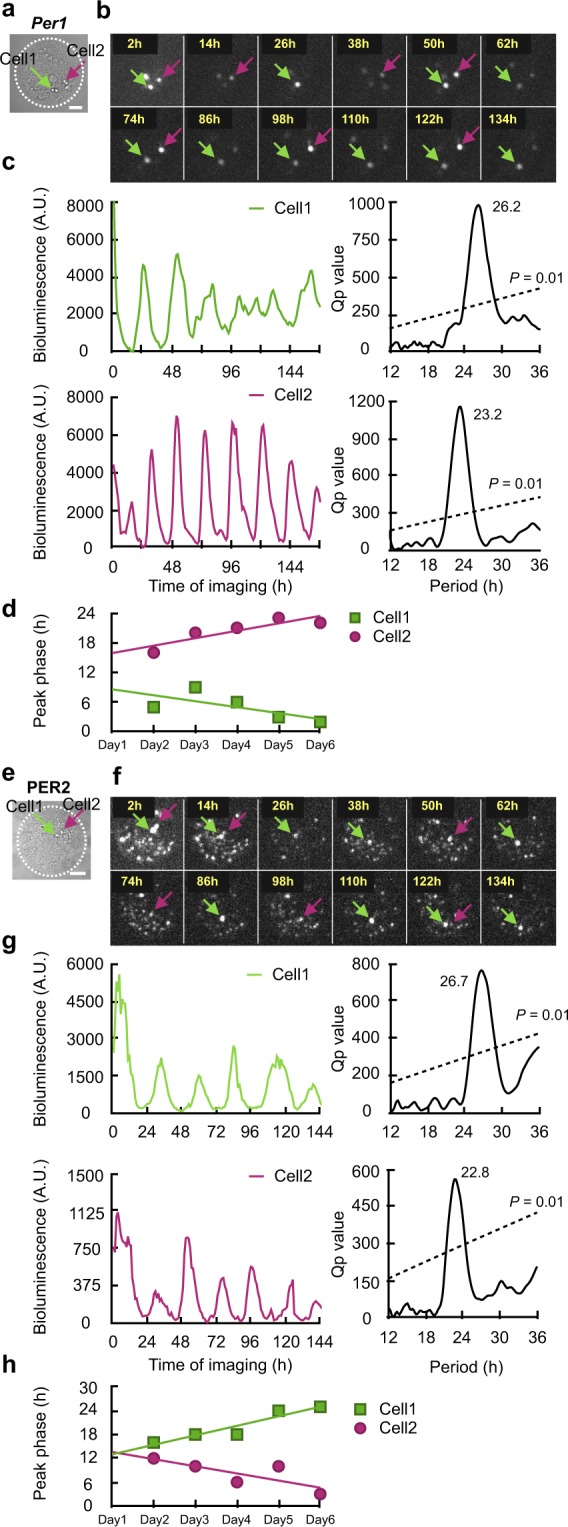
Desynchronization of circadian rhythm between two single SCN neurons on the same microisland. (**a**) Bright-field photomicrograph of two single *Per1* neurons on the same microisland. Arrows indicate two neurons (Cell1, green; Cell2, red). The border of the microisland is indicated by a white dotted circle. Scale bar shows 100 µm. (**b**) Time-lapse images of *Per1-luc* bioluminescence at every 12 hr from the same neurons as in (**a**). (**c**) Circadian rhythm of *Per1-luc* expression in the same neurons as in (**b**) (left) and Chi-square periodogram (right). (**d**) Changes in the peak phases of both circadian rhythms in the course of culture. Solid and broken lines indicate the linear regression lines fitted to the acrophases of two circadian rhythms. (**e**) Bright-field photomicrograph of two single PER2 neurons on the same microisland. Arrows indicate two neurons (Cell1, green; Cell2, red). The border of the microisland is indicated by a white dotted circle. Scale bar shows 100 µm. (**f**) Time-lapse images of PER2::LUC bioluminescence at every 12 hr from the same neurons as in (**e**). (**g**) Circadian rhythm of PER2::LUC expression in the same neurons as in (**f**) (left) and Chi-square periodogram (right). (**h**) Changes in the acrophases of both circadian rhythms in the course of culture. Solid and broken lines indicate the linear regression lines fitted to the acrophases of two circadian rhythms.

A solitary neuron with the PER2::LUC reporter (PER2 neuron) was identified in 10 islands of 12 examined. The islands were also made by collagen spraying method. Among them, a significant circadian rhythm was detected in 7 solitary neurons (70%) by chi-square periodogram (Fig. 2 and Supplementary Fig. 2b). The circadian period was ranging from 22.7 h to 29.2 h. The mean circadian period was 25.9 ± 2.0 h (Fig. 2g). The variability of cycle was 3.2 ± 2.4 h and the damping ratio was 1.4 ± 1.2. One island contained two neurons, both of which were circadian rhythm positive (Fig. 3) and one island contained three neurons, all of which were circadian rhythm positive (Supplementary Fig. 3).

The circadian rhythm in a solitary neuron was quasi-sinusoidal in shape similar to the shape of a single neuron in the SCN slice^28,32,36^. In some neurons, small spikey noises were superimposed on the circadian rhythms (Figs. 1, 2 and Supplementary Fig. 2).

### Desynchronized circadian rhythms of SCN neurons on the same island

Two *Per1* neurons were detected on one island (Fig. 3a–d). These neurons show significant circadian rhythms, which were not synchronized to each other. The circadian rhythm in a cell (Cell1) showed a period of 26.2 h, whereas the other (Cell2) showed a period of 23.2 h (Fig. 3c). Similarly, two PER2 neurons were located on the same island (Fig. 3e–h), the circadian period was 26.7 h for one cell (Cell1), and 22.8 for the other (Cell2). The plots of circadian peak phases with a regression line demonstrate that the phase-angle difference of two circadian rhythms on the same microisland was increasing with a course of culturing, indicating internal desynchronization between the two rhythms (Fig. 3d,h). The other example of desynchronization in three PER2 neurons was demonstrated in Supplementary Fig. 3. The circadian period was 30.2, 23.2 and 24.3 h, respectively. In this and the previous cases, the circadian peaks were located on a similar phase on day 1, suggesting that desynchronization began to occur after culturing (Supplementary Fig. 3).

### Circadian PER2::LUC rhythms in glial cells

**Figure 4:**
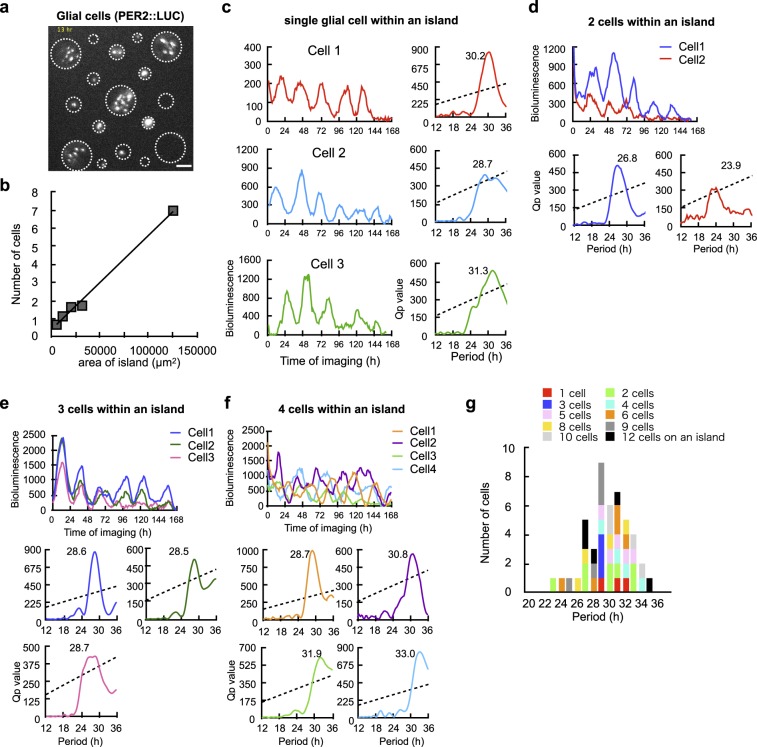
Circadian rhythms in PER2::LUC expression in glial cells. (**a**) Bioluminescent image of PER2 glial cells on various sizes of microisland in the same culture dish. (**b**) Correlation between the size of island and the number of cells locating on it. A strong positive correlation was detected (*n* = 5, R^2^ = 0.994). (**c**) Circadian PER2::LUC rhythms in three solitary glial cells on three different microislands (left) and Chi-square periodograms of each rhythm (right). (**d**) Circadian PER2::LUC rhythms in two glial cells on the same island (upper) and chi-square periodograms of each rhythm (lower). Different colors indicate different cells. (**e**) Circadian PER2::LUC rhythms in three glial cells on the same island (upper) and Chi-square periodograms of each rhythm (lower). Different colors indicate different cells. (**f**) Circadian PER2::LUC rhythms in four glial cells on the same island (upper) and chi-square periodograms of each rhythm (lower). Different colors indicate different cells. (**g**) Distribution of circadian periods in glial cells. Different color columns indicate the number of cells on one microisland. One cell represents each island. The mean circadian period was 29.3 ± 2.6 h (*n* = 46). There was no significant effect of cell abundance on the circadian period.

In order to apply the photolithographic microfabrication method to solitary single cell cultures, we obtained the optimal size of microisland in a preliminary experiment. Glial cells carrying the PER2::LUC reporter were isolated from the cerebral cortex and dispersed to seed on islands of different sizes (40, 60, 80, 100, 200 μm in diameter) (Fig. 4a,b). The mean number of cells located on an island was positively correlated with the island size (*n* = 5, R^2^ = 0.994) and was close to one in the islands of 60 µm (1.13). The mean number in the islands of 40 μm, 80 μm, 100 μm and 200 μm was 0.67, 1.64, 1.72 and 6.96, respectively. To confirm the viability of neurons in these islands, the SCN neurons were seeded on the same sizes of island (Supplementary Fig. 4). The neurons in a 40 and 60 µm islands failed to extend the axon and dendrites probably because of space limitation. By contrast, the axon and dendrites were well extended in the islands of 80 µm and 100 µm, the cell density of which was 0.326 × 10^−3^/µm^2^ and 0.219 × 10^−3^/µm^2^, respectively. Although the mean cell numbers in these islands were larger than one (1.64 and 1.72), we selected the island of 100 µm as the suitable size for solitary neuron culture.

In three culture dishes examined, a single or multiple glial cells were detected in a microisland (Fig. 4c–f). Forty-six cells showed significant circadian PER2 rhythms with periods ranging from 22.8 h to 34.2 h (29.2 ± 2.5 h) (Fig. 4g). Among them, three solitary glial cells showed the circadian rhythm with the period of 30.2, 28.7 and 31.3 h, respectively (all came from either a 60 µm or an 80 µm island) (Fig. 4c). When more than one glial cell was detected in an island, their circadian rhythms were mostly desynchronized to each other showing different circadian period, regardless of the island sizes examined (Fig. 4d–f). There was no systematic effect of cell abundance on the circadian period (Fig. 4g). Glial cells within the same island were all rhythmic regardless of the number of cells at least up to pentad cells.

### Circadian rhythms in intracellular Ca^2+^ in a solitary neuron with and without co-habitant glial cells

By means of a microisland of 100 µm in diameter, we were able to monitor the circadian intracellular Ca^2+^ rhythm of a single neuron continuously for up to 7 days.

**Figure 5:**
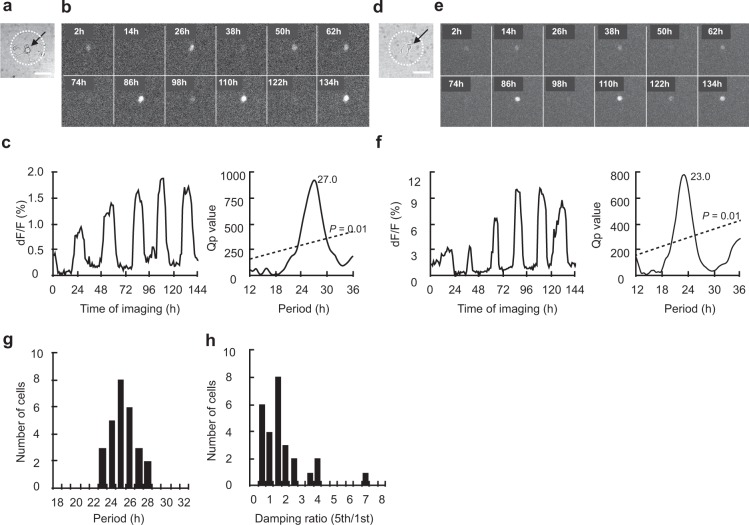
Circadian rhythms in intracellular Ca^2+^ in a solitary SCN neuron on microisland without co-habitant glial cells. (**a**) Bright-field photomicrograph of a solitary neuron on a microisland. The border of the microisland is indicated by a white dotted circle. Scale bar shows 50 µm. (**b**) Time-lapse images of Ca^2+^ fluorescence at every 12 hr from the same neuron as in (**a**). (**c**) Circadian rhythm of intracellular Ca^2+^ in the same neuron as in (**b**) (left) and Chi-square periodogram (right). (**d**) Bright-field photomicrograph of another solitary neuron. (**e**) Time-lapse images of fluorescence from the same neuron as in (**d**). (**f**) Circadian rhythm of intracellular Ca^2+^ in the same neuron as in (**e**) (left) and Chi-square periodogram (right). (**g**) Distribution of circadian period in solitary neurons (*n* = 27) and (**h**) of damping ratio.

**Figure 6:**
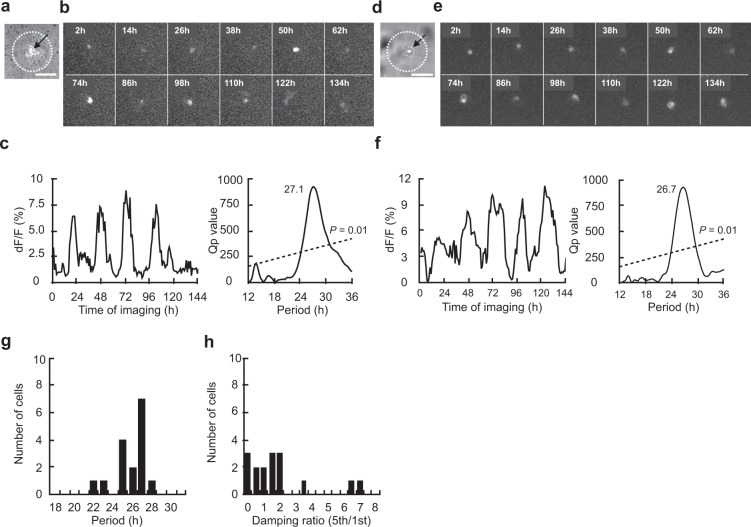
Circadian rhythms in intracellular Ca^2+^ in a solitary SCN neuron on microisland with co-habitant glial cells. (**a**) Bright-field photomicrograph of a solitary neuron on a microisland. The border of the microisland is indicated by a white dotted circle. Scale bar shows 50 µm. (**b**) Time-lapse images of Ca^2+^ fluorescence at every 12 hr from the same neuron as in (**a**). (**c**) Circadian rhythm of intracellular Ca^2+^ in the same neuron as in (**b**) (left) and Chi-square periodogram (right). (**d**) Bright-field photomicrograph of another solitary neuron. (**e**) Time-lapse images of fluorescence from the same solitary neuron as in (**d**). (**f**) Circadian rhythm of intracellular Ca^2+^ in the same neuron as in (**e**) (left) and Chi-square periodogram (right). (**g**) Distribution of circadian period in solitary neurons (*n* = 16) and (**h**) of damping ratio.

For culturing a solitary neuron, we used two slightly different methods, one with a pre-culture of dispersed cortical glial cells on microislands (7 dishes) and the other without pre-culture (10 dishes). Since the ratio of circadian rhythm appearance was not significantly different (*P* = 0.44) between the two methods (24.3% with cortical glial cells and 25.9% without them), we pooled the results. Among 96 islands in 17 dishes examined, 32 islands (12 dishes) contained a solitary neuron without glial cells (Fig. 5) and 64 islands (17 dishes) contained a single neuron with glial cells in the same island (Fig. 6). Twenty-seven single neurons without glial cells (84.4%) showed significant circadian rhythms, whereas only 16 solitary neurons with glial cells (25.0%) showed circadian rhythms. The ratio of rhythm positive neurons was significantly lower with co-habitant glial cells than without them (chi-square test, *P* < 0.01). The circadian period was ranged from 22.7 h to 27.5 h with the mean period of 24.7 ± 1.3 h without glial cells and ranged from 21.8 h to 27.1 h with the mean period of 25.3 ± 1.5 h when cohabitating with glial cells. The mean circadian period was not significantly different (*P* = 0.19). The variability of cycle intervals in terms of SD was 1.6 ± 0.8 and the damping ratio was 2.0 ± 1.4 without glial cells, whereas the variability was 2.0 ± 1.2 and the damping ratio was 2.1 ± 2.0 with glial cells. Both parameters were not significantly different between them (*P* = 0.92). Circadian rhythm profiles in other solitary neurons were illustrated in Supplementary Figs. 6–9.

The circadian rhythm of intracellular Ca^2+^ in a solitary neuron was rather rectangular than quasi-sinusoidal in shape similar to the shape of circadian rhythm in electric activity^37^. The intracellular Ca^2+^ level in a circadian cycle steeply increased or decreased, showing a plateau with small spiky fluctuation at its highest level, typically in Fig. 6f.

## Discussion

Circadian rhythms in clock gene (*Per1* and PER2) expression and intracellular Ca^2+^ were detected in a large fraction of solitary SCN neurons cultured on a microisland of 100–200 μm in diameter. To our knowledge, this is the first attempt of systematic monitoring of the circadian rhythm in a fully and physically isolated solitary SCN neuron without synaptic connections or gap junctions between other cells. The circadian periods were slightly longer than 24 hr on average. In terms of the damping ratio and cycle-to-cycle variability, the rhythm stability was kept unchanged or even became better on microisland, and as robust as those in slice culture previously reported from our laboratory^20,37^. When two neurons were cultured on the same microisland, they are not synchronized to each other. Interestingly, as for the circadian rhythm in intracellular Ca^2+^, the ratio of rhythm positive neurons was significantly lower when SCN neurons are co-habitant with glial cells than when without it. These features of circadian rhythms in solitary neurons are marked contrast with those reported previously in selected dispersed neurons^3^. Approximately 60% of the solitary neurons in *Per1* expression, more than 70% of the solitary neurons in PER2 expression showed circadian rhythm and more than 80% showed circadian rhythm in intracellular Ca^2+^. The cell density of the present condition was identical to 62–195 cells/mm^2^ in a conventional culture dish. Previously, the expression of circadian PER2::LUC rhythm in the SCN neuron decreased when the cell density in dispersed cell culture was reduced. The ratio of rhythm-positive neurons was 80% when cultured at the concentration of 10,000 cells/mm^2^ and 30–50% when cultured at the concentration of 3,000–5000 cells/mm^2,3^. The ratio was reduced to less than 16% at the concentration of 100 cells/mm^2^, which was almost equivalent to the present study^3^. The findings are interpreted to indicate that only a fraction of SCN neurons is an intrinsic oscillating cell and other neurons are reinforced or driven through the neural networks. In the present study, a relatively large fraction (70.0% in PER2 expression and 84.4% in intracellular Ca^2+^) of solitary SCN neuron showed significant circadian rhythms, suggesting most neurons in the SCN, if not all, are intrinsic oscillating cells.

The shape of circadian rhythms seemed to be associated with variables, or functions measured; quasi-sinusoidal in clock genes and rather rectangular in intracellular Ca^2+^ level. Quasi-sinusoidal circadian rhythm of a solitary neuron in the present study was a marked contrast with noisy and sloppy circadian rhythms previously observed in a fully isolated SCN neuron in dispersed cell culture^3^. It is however not known whether or not the individual difference in shape was associated with the robustness of a solitary neuron in culture.

The difference in shape between clock gene expression and intracellular Ca^2+^ level could be due to the reporter system used but more likely to the functional differences between the clock gene and intracellular Ca^2+^. The clock gene expression is a process in the autofeedback loop, while intracellular Ca^2+^ may also change instantaneously in response to external stimuli. The neuronal activity rhythm of a single SCN cell is quasi-rectangular in shape on a multiple electrode dish^25,26^. The circadian rhythm in intracellular Ca^2+^ may be sculptured by an intrinsic circadian oscillation and external inputs through the neural connections.

In the present study, a solitary neuron on a microisland had no physical contact through synapse or gap junction with other cells. Besides the neural networks, humoral factors such as VIP and AVP are known to synchronize neuronal circadian rhythms in the SCN^31–33,38^. Co-culture of wild type neonatal SCN slice is demonstrated to rescue the circadian rhythm in an arrhythmic SCN slice from adult *Cry1*/*Cry2* deficient mice^37^. In the present study, more than one neuron in the same island showed desynchronized circadian rhythms with different periods. The finding suggests that the humoral factor(s) secreted from the neighboring neurons, if any, is not effective enough to synchronize the cells under the present experimental conditions. A failure of synchronization could be due to a lack of secretion of an active mediator, of a responsible receptor, or of a small diffusion constant. In any case, the finding indicates the intrinsic circadian rhythm in a single SCN neuron persists for at least several cycles without any interaction with other cells.

TTX is known to desynchronize the circadian rhythms in single SCN neurons in slice culture, indicating that the neural network is important for the mutual synchronization of oscillating neurons^20,28,37^. TTX is also reported to attenuate the amplitude of bioluminescence rhythms and abolish the rhythmicity in approximately half of neurons examined^28^. On the other hand, TTX application in the SCN did not disrupt the circadian oscillation *in vivo*^30^. This inconsistency suggests us the neuronal network in the SCN is less potent in the slice culture than in the whole.

To our surprise, the ratio of circadian Ca^2+^ rhythm positive solitary neurons was significantly reduced when glial cells were cohabitated in their islands. Glial cells show intrinsic circadian rhythms without the SCN neurons^35^ and modify the circadian oscillation of the neurons^8–11^. In the present study, the period of circadian PER2 rhythm in single glial cells was slightly longer than that of SCN neurons, which was inconsistent with previous reports^39^. In addition, the circadian rhythms of glial cells were mostly desynchronized in the present conditions. The presence of glial cells may compromise the circadian Ca^2+^ rhythm in neurons. This may not happen in the intact SCN. The reason for this compromise is unknown but likely related to cell ratio. The ratio of glial cells to the SCN neurons is much larger in the present microisland (larger than 1.0) than *in vivo* where the ratio is ca. 0.3^40^. The mechanism of interference is not known but several molecules such as ATP^34^, GABA^11,41^, Glutamate^9^ and also intracellular Ca^2+^ ^42^ are possible mediators. Further studies are needed to clarify the mechanism.

In conclusion, a relatively large fraction of solitary SCN neurons is an intrinsically oscillating cell. The circadian rhythms in solitary neurons are comparable with those of a single neuron in the cultured SCN slice in terms of the damping ratio and cycle-to-cycle stability. Glial cells interfere with the circadian rhythm in intracellular Ca^2+^ in a solitary SCN neuron, indicating suppressive influence from glial cells on the neurons under a microenvironment with an abundance of glial cells over neurons.

## Methods

### Animals

For monitoring of clock gene expression *ex vivo*, C57BL/6J mice carrying a bioluminescence *Period1* (*Per1-luc*)^43^ and PERIOD2 (PER2::LUC)^44^ reporter were bred and reared in our animal quarters where environmental conditions were kept constant (temperature: 22 ± 2 °C, humidity: 60 ± 5%, 12/12-h light–dark with lights-on from 06:00 to 18:00 h). Light intensity during the light phase was around 100 lx at the cage surface. For measuring the intracellular Ca^2+^ level, mice of 15th-day pregnancy (C57BL/6J, Clea Japan) were purchased and reared in the above-mentioned animal quarter. They had free access to mouse chow (Oriental Yeast Co, Tokyo, Japan) and tap water. New-born (postnatal day 1 to day 3: P1–3) mice were used for *ex vivo* experiments. All experiments were conducted in compliance with the rules and regulations established by the Animal Care and Use Committee of Hokkaido University under the ethical permission of the Animal Research Committee of Hokkaido University (Approval No. 15-0153).

### Preparation of microisland in the culture dish

Two different methods were introduced for culturing a single neuron or glial cell physically isolated from other cells; Collagen Spraying Method and Photolithographic Microfabrication Method. The former method was used for monitoring clock gene expression in the SCN neuron and the later for intracellular Ca^2+^ level in the SCN neuron and glial cells from the cerebral cortex.

#### Collagen spraying method

In order to use inverted microscopy for imaging, a culture dish was custom-made using a 35 mm petri dish. A hole (12 mm in diameter) was made in the bottom of a petri dish and a cover glass was attached on the outer bottom surface with silicone elastomer (Sylgard 184, Dow Corning). The inside of a dish including a closed hole was coated with 2.5% agarose solution of low-melting-point by spin coater to avoid cell attachment. 0.05% solution of rat-tail collagen (Type I-A, Sigma-Aldrich; dissolved in 0.15 M acetic acid) was sprayed on the agarose-coated surface of a dish to make a number of microislands for culturing solitary neurons^45^. The size of microislands varied from 100 to 300 μm in diameter. The culture dish was dried and sterilized with ultraviolet (UV) radiation in a clean bench, and then washed 3 times with 0.1 M phosphate buffered saline (PBS) before cell culture.

#### Photolithographic microfabrication method

A large number of microislands were made on a Poly(2-methacryloyloxyethyl phosphorylcholine) (MPC) -*co*-3-methacryloyloxyproryl triethoxysilane (MPTS) (i.e., MPC polymer)^46^ which was coated on a cover glass (20 × 20 mm, No.2 thickness, Matsunami Glass Ind., Ltd.), where a solitary cell is unable to adhere^47^. MPC solution was spun at 2000 rpm for 30 s onto a cover glass and dried in a chamber with an ethanol atmosphere at room temperature for 20 min to make the polymer layer uniformly. Then, they were baked at 70 °C for 4 h to covalently graft MPC polymer to the cover glass. Subsequently, a 1 µm-thick parylene film (Parylene-C; Specialty Coating Systems, USA) was deposited onto the MPC-coated layer and photoresist layer was coated on it (Supplementary Fig. 1b-(I)). By a standard photolithographic technology, small wells with a diameter of 40 to 200 µm were made (Supplementary Fig. 1b-(II–IV) as follows. The MPC-coated cover glass was exposed to ultraviolet light through a photomask (Supplementary Fig. 1b-(II)). The photoresist layer at the exposed sites was cleaned off (Supplementary Fig. 1b-(III)). Both parylene film and MPC polymer were etched away with O_2_ plasma (10 ml/min, 25 W for 8 min) (Supplementary Fig. 1b-(IV)). The micropatterned cover glass was stored at the desiccator. When used, a micropatterned cover glass was attached on the bottom of the petri dish with a hole and sterilized by UV light radiation for 1 hr. Human fibronectin solution (10 µg/ml in PBS(−)) was incubated in micropatterned small wells for 30 min to increase cell adhesion. Immediately before culturing, a parylene film was peeled off from the micropatterned cover glass (Supplementary Fig. 1b-(V)).

### Solitary cell culture

#### SCN neurons

In one series of experiment, eight to 10 newborn (P1–P3) mice were anesthetized on ice and the brains were quickly removed to place in ice-cold Hank’s balanced salt solution (Hank’s solution). The brain was cut coronally at 300 µm thickness with tissue chopper and the SCN region was dissected. Collected SCN tissues were dissociated using papain (13~20 units/ml; Worthington Biochem. Corp.) and DNase I (1 mg/ml) at 37 °C for 20 min. The dissociated cells were washed with 50% horse serum and collected by spin-down at 1,500 rpm for 5 min. The cells were dispersed using pipette in Dulbecco’s modified Eagle’s medium (DMEM) containing 10% fetal bovine serum (FBS) and 1% penicillin/streptomycin. Cell suspension was filtrated with cell strainer (ø 40 µm; Falcon, Thermo Fisher Scientific). The 200 µl of filtrated cell suspension was placed onto the center hole (ø 1.2 mm) of culture dish at a density of 3.5–11.0 × 10^4^ cells/ml (5.8 ± 3.2 × 10^4^ cells/ml). One day before seeding, the dispersed cortical glial cells were pre-cultured in the culture dish. One hour after seeding, the dish was washed with culture medium to remove excessive cells suspended in the dish. Afterward, the culture medium was exchanged twice a week until imaging experiment.

#### Glial cells

Two newborn (P2–P4) mice carrying PER2::LUC reporter were anesthetized on ice and the removed brains were placed in ice-cold Hank’s solution. The brain was bisected at midline, and the bilateral cerebral cortices were dissected. Pia mater was removed from the cortex under the binocular microscope, and the cortex was chopped into small pieces using two surgical knives. The tissues were dissociated with 0.25% trypsin solution containing 0.5 mM EDTA at 37 °C for 15 min. The dissociated cells were washed with 50% house serum and dispersed by pipette in DMEM containing FBS and 1% penicillin/streptomycin. The cells were seeded in a T-75 culture flask and cultured until confluent growth. Afterward, the cells were dissociated again with trypsin/EDTA solution to exclude neurons and other tissues. The cells were placed onto the collagen dots and the micropatterns at the density 0.1–1.0 × 10^5^ cells/ml. Two to three days later, the culture medium was exchanged twice a week.

### Bioluminescent and fluorescent imaging

#### Per1-luc and PER2::LUC imaging

Bioluminescent imaging was carried out as reported previously (Ono *et al*.^36^). Before the imaging was started, the culture medium was exchanged to that containing 5% FBS, 2% B27 supplement, 2.5 mM L-glutamine, 15 mM HEPES, 0.1 mM D-luciferin potassium and 1% penicillin/streptomycin in DMEM/Nutrient Mixture F12. Bioluminescence from each cell was captured by Luminoview (LV200, Olympus) equipped with an EMCCD camera (512 × 512 pixels; ImagEM, Hamamatsu Photonics or iXon Ultra 897, Andor Tech.) cooled at −80 °C, dry objectives (40×, 0.9 NA, UPLSAPO; Olympus) and 0.2× relay lens. Imaging area is 1024 × 1024 μm^2^ which covers one or two islands at same time in the collagen spray method. The measurement was done every 60 min with an exposure time of 59 min for at least consecutive 7 days. In a single experiment, bioluminescent measurement was performed in 1 dish. During measurement, the culture medium was not exchanged.

#### Intracellular Ca^2+^ imaging

A genetically-encoded Ca^2+^ sensor, GCaMP6s, was transduced using adeno-associated virus (AAV, serotype 2/1) was carried out as reported previously^20^. After aspirating culture medium from a dish, 5 µl of AAV encoding hSyn1-GCaMP6s (1.31 × 10^13^ GC/ml; produced at the University of Pennsylvania Gene Therapy Program Vector Core) solution was dropped on the cells adhered on microislands one or two days after the start of culture. In 7 culture dishes, the dispersed cortical glial cells were pre-cultured, and in 10 dishes the pre-cultured was not performed. The culture dish was incubated at 37 °C, 5% CO_2_ incubator at 100% humidity for 1 h, and then added with 2 ml of fresh culture medium. Ten days after infection, time-lapse calcium imaging for fluorescent signals was carried out for 6–7 days.

Time-lapse confocal imaging was conducted with an exposure time of 1 sec at 60 min intervals. The imaging system is composed of an EM-CCD camera (1024 × 1024 pixels, iXon3; Andor Technology), inverted microscope (Ti-E; Nikon), dry objectives (20×, 0.75 NA, Plan Apo VC; Nikon), spinning disk confocal system (X-Light; CREST OPTICS), box incubator (TIXHB; Tokai-hit), and MetaMorph software (Molecular Devices). CaMP6s was excited by cyan color (475/28 nm) with the LED light source (Spectra X Light Engine; Lumencor Inc.) and the fluorescence was visualized with 495-nm dichroic mirror and 550/49-nm emission filters (Semlock). Imaging area is 1664 × 1664 μm^2^ which cover 25 islands at same time. By using computer-controlled XY stages, we searched for solitary neurons in 900 microislands of a dish. Before measurement, culture medium was changed to that containing 5% FBS, 2% B27 supplement and 1% penicillin/streptomycin. The dishes were sealed with O_2_-permeable filters (membrane kit, High Sens; YSI). All experiments were performed at 36.5 °C and 5% CO_2_ on the stage of inverted microscope. In a single experiment, the measurement was simultaneously performed using 1 to 4 dishes. And the same experiment was repeated 5 times. The cell condition was monitored by the bright field images of cultured neurons and by the calcium signals in individual neurons. If we detected noticeable cell damage during the recording, such as swelling of cell and abrupt/large calcium increase which was usually followed by the permanent disappearance of fluorescence signals (the sign of cell death), we excluded the data for the analysis.

### Immunocytochemistry

The neurons and glial cells were immunocytochemically distinguished after fluorescence imaging. Antibodies for gamma amino butyric acid (GABA) and glial fibrillary acidic protein (GFAP) were used for identification of the neurons and glial cells, respectively. Cells were fixed with 4% paraformaldehyde (PFA) for 30 min. Fixed cells were immunized with rabbit anti-GABA polyclonal antibody (1:1000 dilution; Millipore) and mouse anti-GFAP monoclonal antibody (1:1000 dilution; Sigma-Aldrich) in PBS including 0.1% Triton X-100 and 1% goat serum for 2 days in 4 °C. Alexa 488-conjugated goat anti-rabbit IgG and Alexa 594-conjugated goat anti-mouse IgG (1:200 dilution; Invitrogen) were used as the second antibody for GABA and GFAP, respectively. GABA- and GFAP-positive fluorescent signals were visualized with inverted fluorescent microscopy (BioRevo, Keyence).

### Identification of neurons and glial cells

The SCN neurons and glial cells in a microisland were easily distinguished by their morphological characteristics such as a smaller but more compact cell body occupied mostly by a nucleus with a limited cytosolic area and an axon and dendrites in the neurons, as compared with a larger and flatter cell body in the glial cells. The cell body of a neuron is also characterized as brighter and more contrasted appearance than that of the glial cell under bright-field microscopy. Immunocytochemical identification using specific markers for SCN neurons (GABA) and glial cells (GFAP) confirmed the cell types after the end of culture (Supplementary Fig. 5).

### Data analysis

Imaging data were analyzed using ImageJ image processing and analysis software. Photometric signals from cells were tracked manually at every one frame, because of cell movement. Bioluminescent signals were expressed as an arbitrary unit (A.U.). The Ca^2+^ level was evaluated as ∆F/F = (F − F_0_)/F_0_, where F is the raw fluorescent signal and F_0_ is a minimum value of the raw signal of the whole series.

The significance of the circadian rhythm was assessed by a chi-squared periodogram written in Matlab script using a record of 144 h for each neuron. Significance level was set with the peak Qp value of *P* < 0.01. The circadian period was examined in the range from 12 to 36 hrs and the rhythmicity outside the range was regarded as non-circadian (arrhythmic). Acrophase of each circadian cycle was determined by fitting each cycle’s data to a sine curve (ClockLab, Actimetrics). Unless otherwise stated, mean values are expressed as the mean ± the standard deviation (SD). A difference between the two group means was statistically tested by unpaired t-test. The probability of occurrence was tested by chi-square test.

## Supplementary information

Supplementary Information

## Data Availability

The data that support the findings of this study are available from the corresponding authors upon reasonable request.

## Code availability

Code is available from the corresponding author upon request.
