# Supplementary material for: Circadian rhythms in Per1, PER2 and Ca2+ of a solitary SCN neuron cultured on a microisland
Source: Sci Rep. 2019 Dec 4;9:18271. doi: 10.1038/s41598-019-54654-5 (PMC6892917; doi:10.1038/s41598-019-54654-5)
Supplement: Supplementary file 1 — Supplementary Information [file 41598_2019_54654_MOESM1_ESM.pdf]

Supplementary information

Circadian rhythms in *Per1*, PER2 and Ca^{2+} of a solitary SCN neuron cultured on a microisland.

Yoshihiro Hirata[1,†] Ryosuke Enoki[1,4,6,7,†,*], Kaori Kuribayashi-Shigetomi[2,3,†], Yoshiaki Oda[4,5,8], Sato Honma[4,5], Ken-ichi Honma[4,5,*]

Author affiliation

1. Photonic Bioimaging Section, Hokkaido University Graduate School of Medicine, Sapporo, Japan
2. Institute for the Advancement of Higher Education, Hokkaido University, Sapporo, Japan
3. Graduate School of Information Science and Technology, Hokkaido University, Sapporo, Japan
4. Department of Chronomedicine, Hokkaido University Graduate School of Medicine, Sapporo, Japan
5. Research and Education Center for Brain Science, Hokkaido University, Sapporo, Japan
6. Present address: Biophotonics Research Group, Exploratory Research Center on Life and Living Systems (ExCELLS), National Institutes of Natural Sciences, Okazaki, Japan
7. Present address: Division of Biophotonics, National Institute for Physiological Sciences, National Institutes of Natural Sciences, Okazaki, Japan
8. Present address: Department of Oral Chrono-Physiology, Unit of Basic Medical Sciences, Graduate School of Biomedical Sciences, Nagasaki University, Nagasaki, Japan

†. These authors contributed equally to this work.

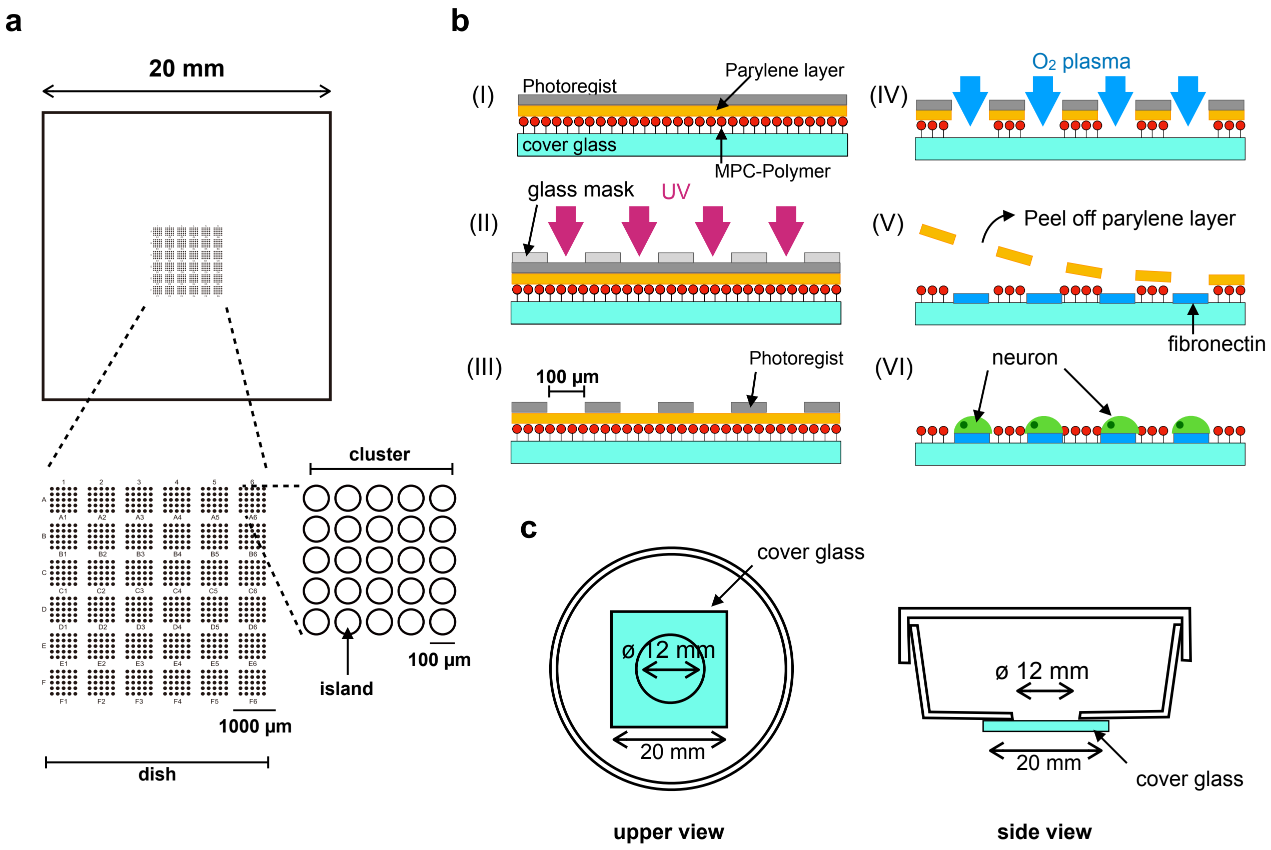

Supplementary Figure 1

Supplementary Figure 1. Schematic diagram of the microisland preparation procedures for collagen spraying and photolithographic microfabrication methods. (a) Design for glass mask making microislands on a cover glass. In this case, 900 microislands of 100 μm diameter were located on the center of glass. A micropatterned cover glass was generated by microfabrication technology. The Cover glass was coated by 2-methacryloyloxyethyl phosphorylcholine (MPC) polymer (I). A thin layer (1μm) of poly(p-xylylene) (Parylene) was formed on the MPC polymer layer. The photoresist was coated on the parylene layer and patterned using a standard photolithographic technique (II&III). The MPC and parylene layers were etched away with the O_2 plasma (IV). Human fibronectin was coated on a micropatterned glass (V). Neurons or glial cells were seeded on microislands (VI). (c) Illustration of a custom-made glass-bottom culture dish.

a *Per1*

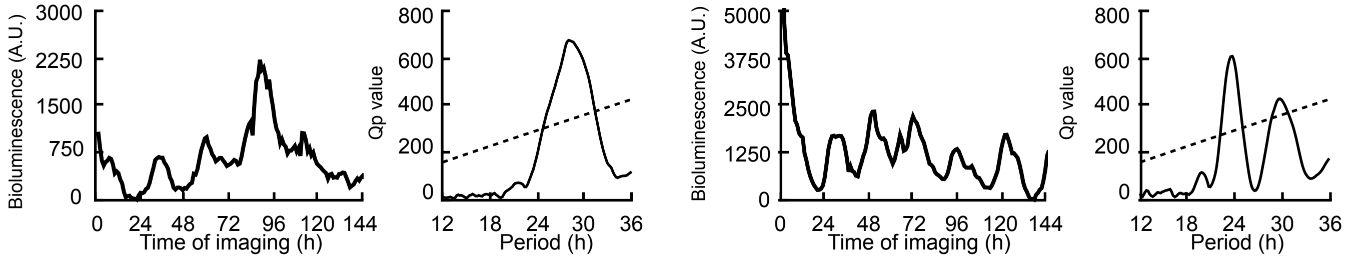

b PER2

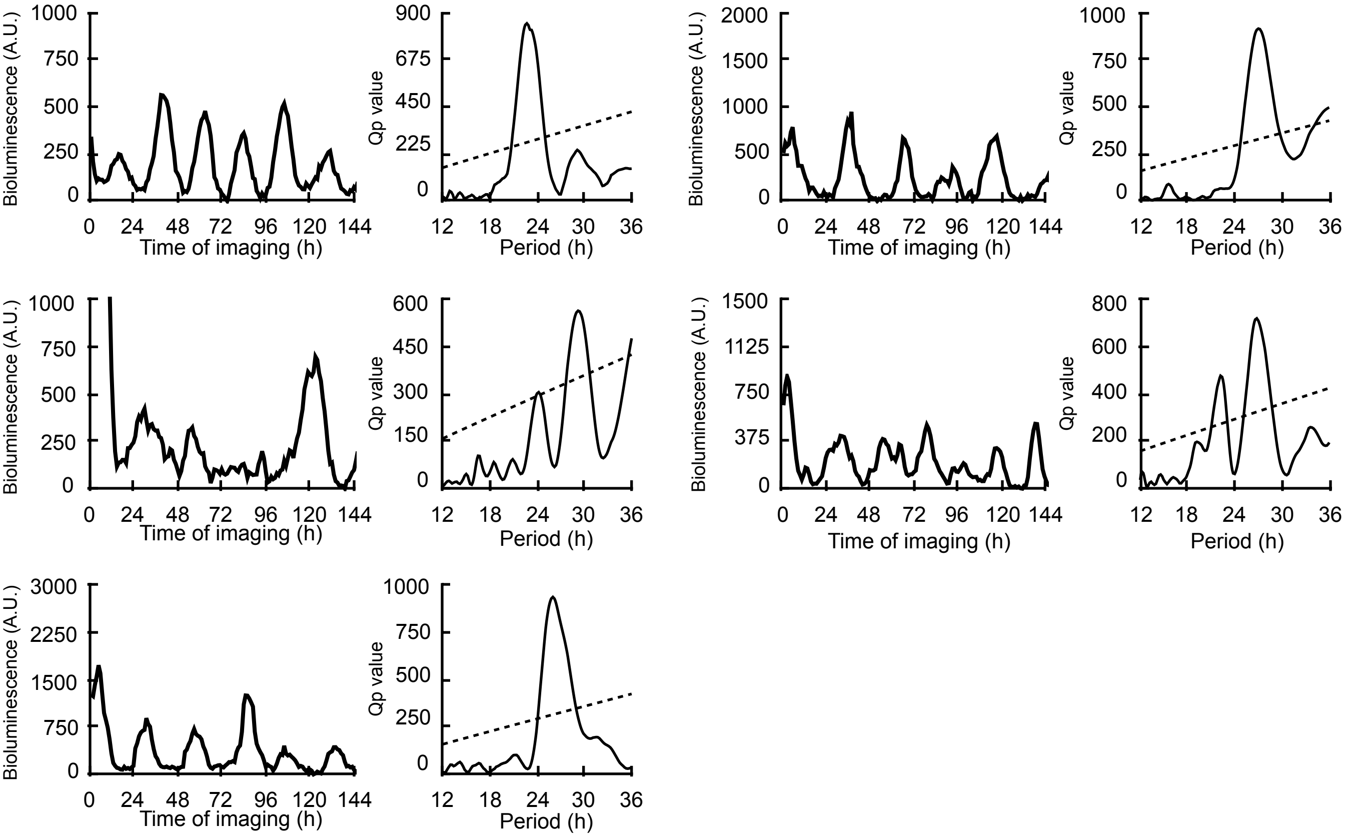

Supplementary Figure 2

Supplementary Figure 2. Circadian rhythm in *Per1-luc* and PER2::LUC in a solitary SCN neuron on a microisland.

(a) Circadian rhythms of *Per1-luc* in other two solitary SCN neurons on different microisland (left) and Chi-square periodogram (right). (b) Circadian rhythms of PER2::LUC in other five solitary SCN neurons on different microisland (left) and Chi-square periodogram (right).

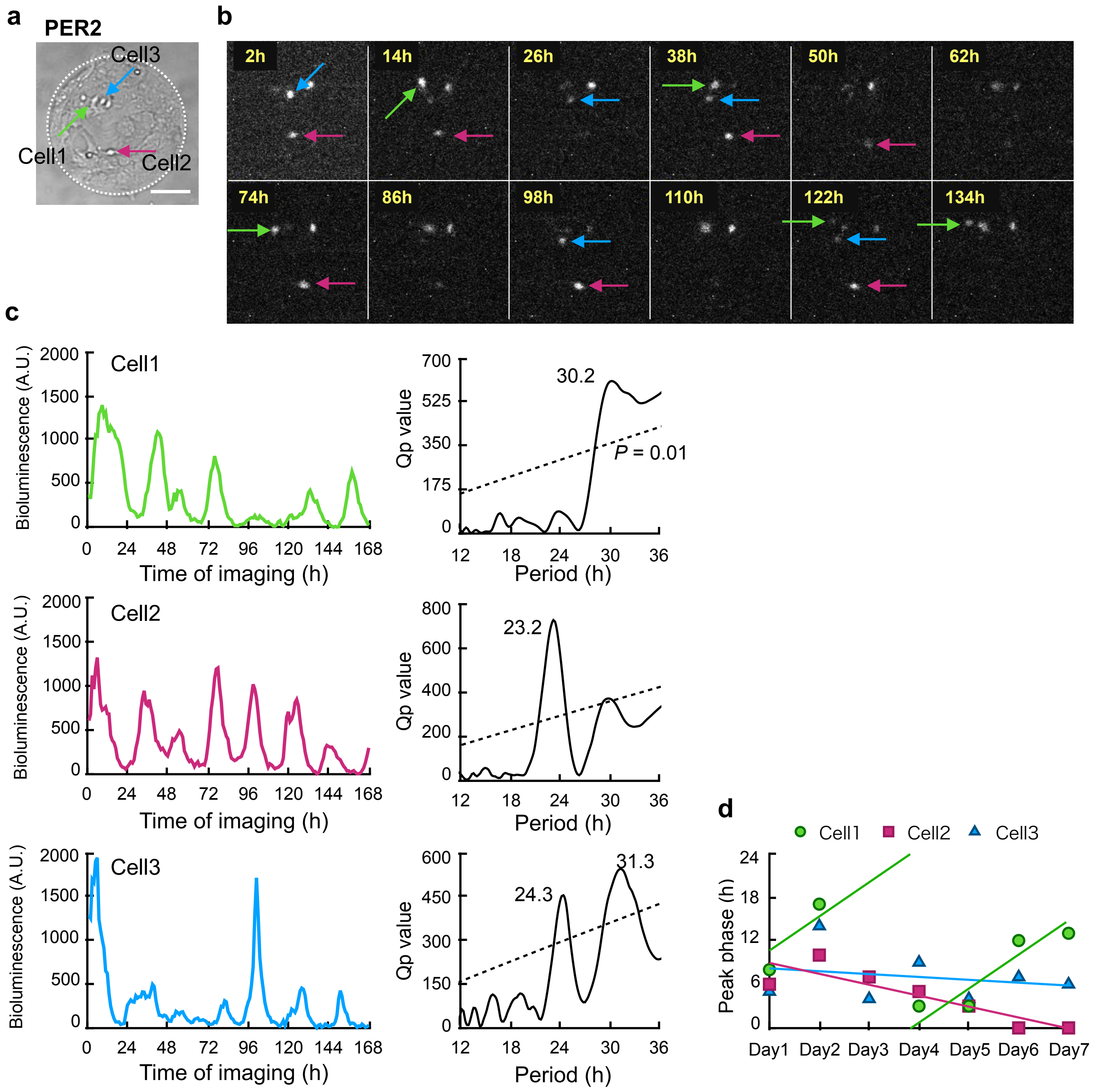

Supplementary Figure 3

Supplementary Figure 3. Desynchronization of circadian PER2::LUC rhythms in three single cells in the same island.

(a) Bright-field photomicrograph of three PER2::LUC neurons on the same microisland. Arrows indicate three neurons (Cell1, green; Cell2, red; Cell3, yellow). The border of the microisland is indicated by a white dotted circle. Scale bar shows 100 µm. (b) Time-lapse images of PER2::LUC bioluminescence at every 12 hr from the same neurons as in (a). (c) Circadian rhythms of PER2::LUC expression in the same neurons as in (b) (left) and Chi-square periodogram (right). (d) Changes in the peak phases of three circadian rhythms in the course of culture. Solid, broken and dotted lines indicate the linear regression lines fitted to the acrophases of three circadian rhythms.

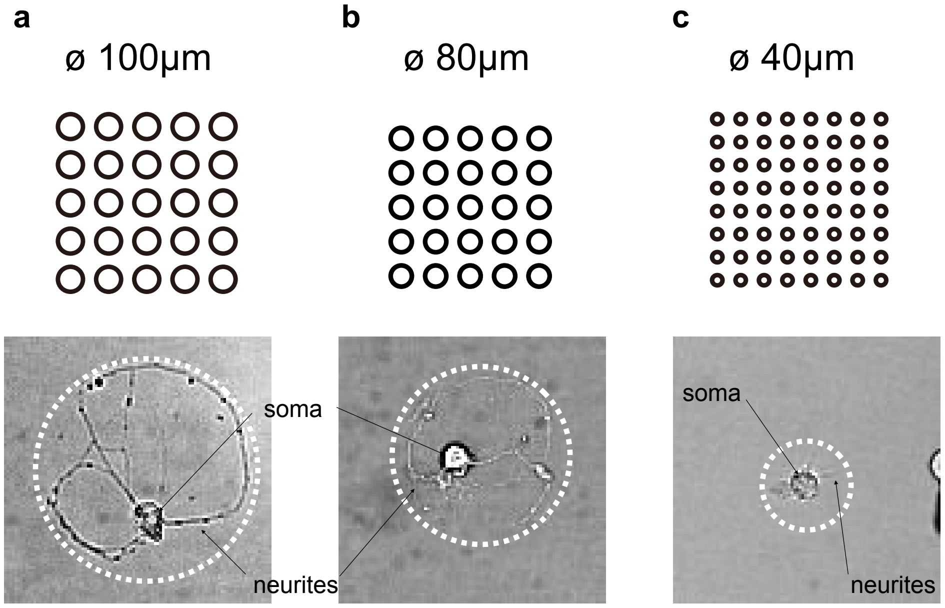

Supplementary Figure 4

Supplementary Figure 4. Photograph of a solitary SCN neuron on a different size of microisland.

Diameter size of 80 to 100 μm microisland was suitable for culturing a solitary neuron. In 40 μm diameter of the microisland, a solitary neuron did not elongate their processes and tended to become apoptotic and die in culture.

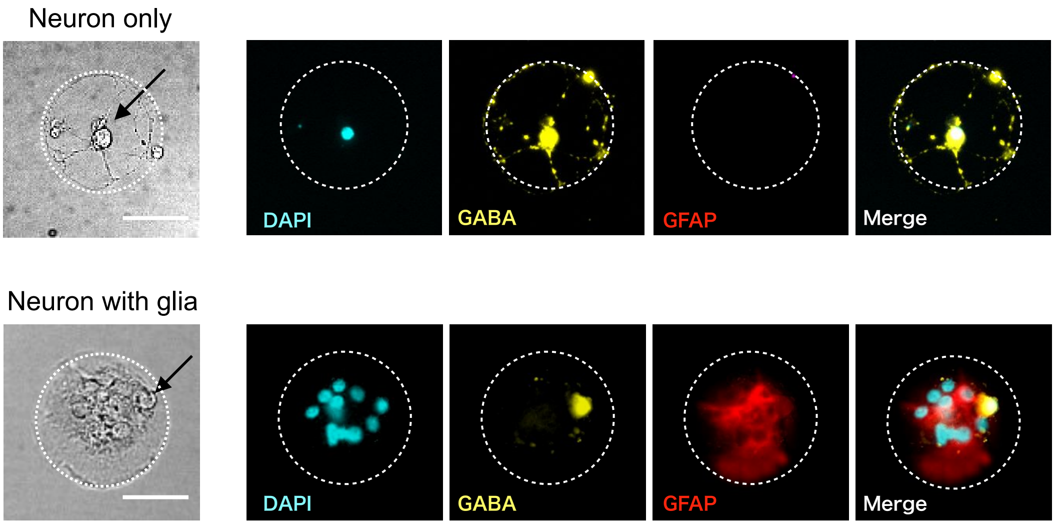

Supplementary Figure 5

Supplementary Figure 5. Immunocytochemical fluorescent images of a solitary SCN neuron with or without co-habitant glial cells on a microisland.

Bright field photomicrograph (far left) and immunocytochemical fluorescent images of DAPI (light blue), GABA (yellow), GFAP (red) and the overlay are illustrated. A yellow arrow in the photomicrograph indicates a solitary SCN neuron. The border of the microisland is indicated by white dotted circles. Scale bar shows 50 μm.

Neuron only (rhythmic)

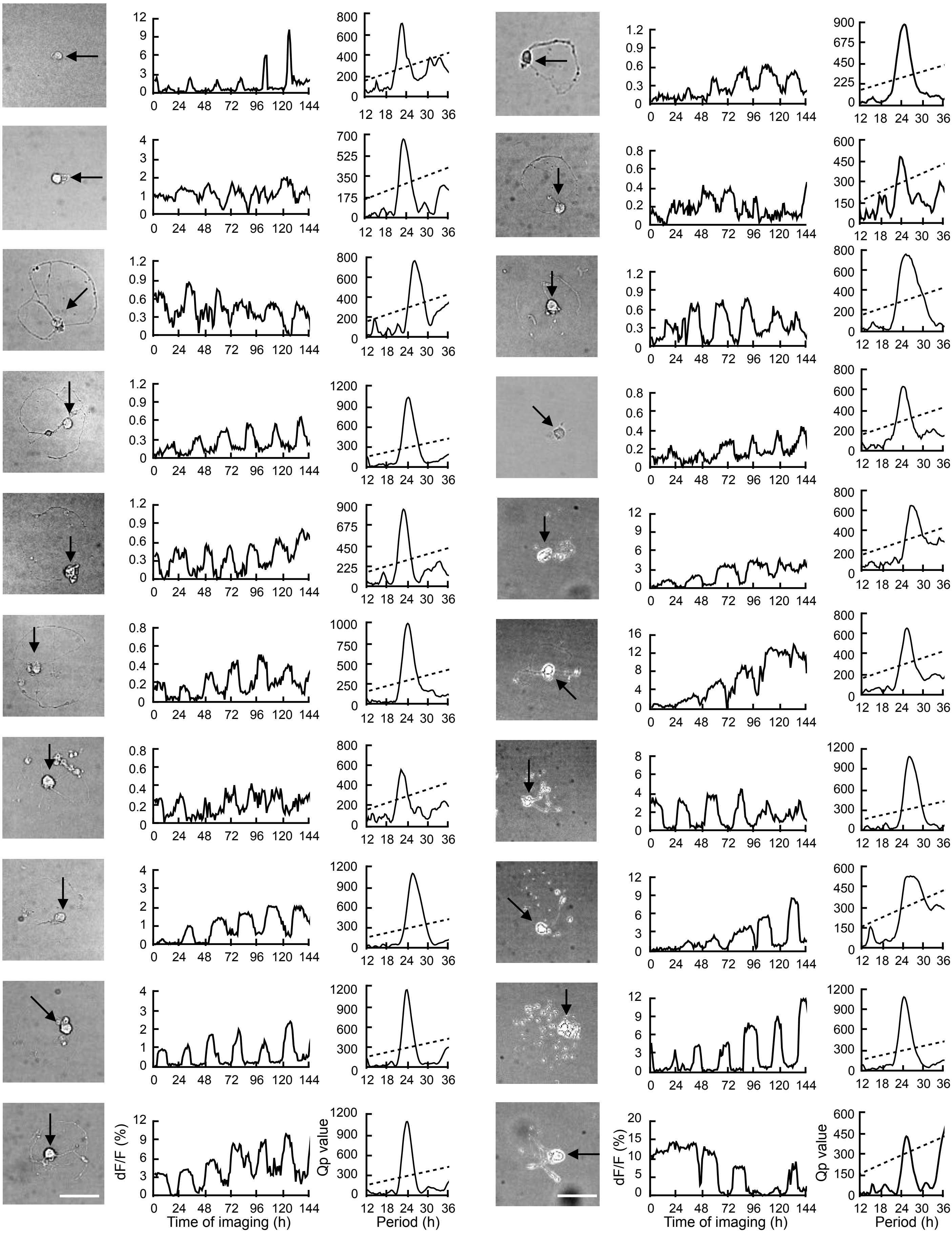

Supplementary Figure 6-1

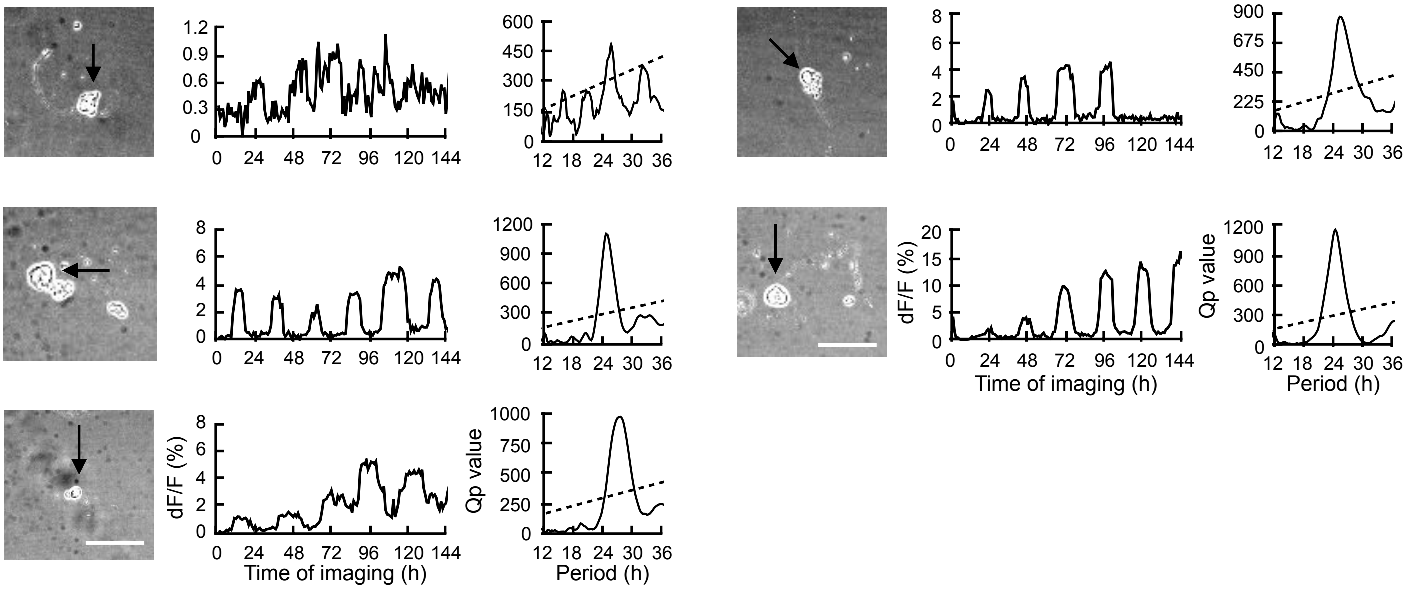

Supplementary Figure 6-2

Supplementary Figure 6. Circadian Ca^{2+} rhythms in solitary SCN neurons without co-habitant glial cells.

Circadian Ca^{2+} rhythm (left) and Chi-square periodogram (right) of solitary SCN neurons other than the results illustrated in Fig.5.

Neuron only (arrhythmic)

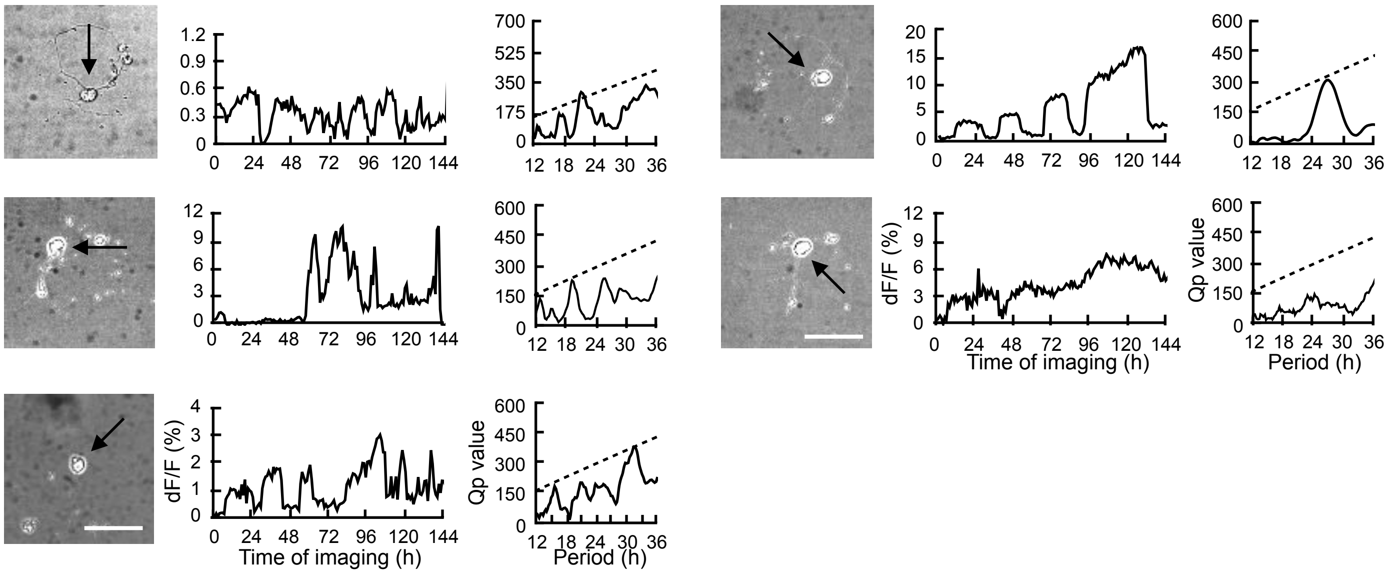

Supplementary Figure 7. Arrhythmic Ca^{2+} expression in solitary SCN neurons without co-habitant glial cells.

Five examples of arrhythmic Ca^{2+} expression in solitary SCN neurons without co-residence of glial cells. Bright-field photomicrograph (left), time courses of fluorescent signals (middle) and Chi-square periodogram (right) are illustrated.

Neuron with glia (rhythmic)

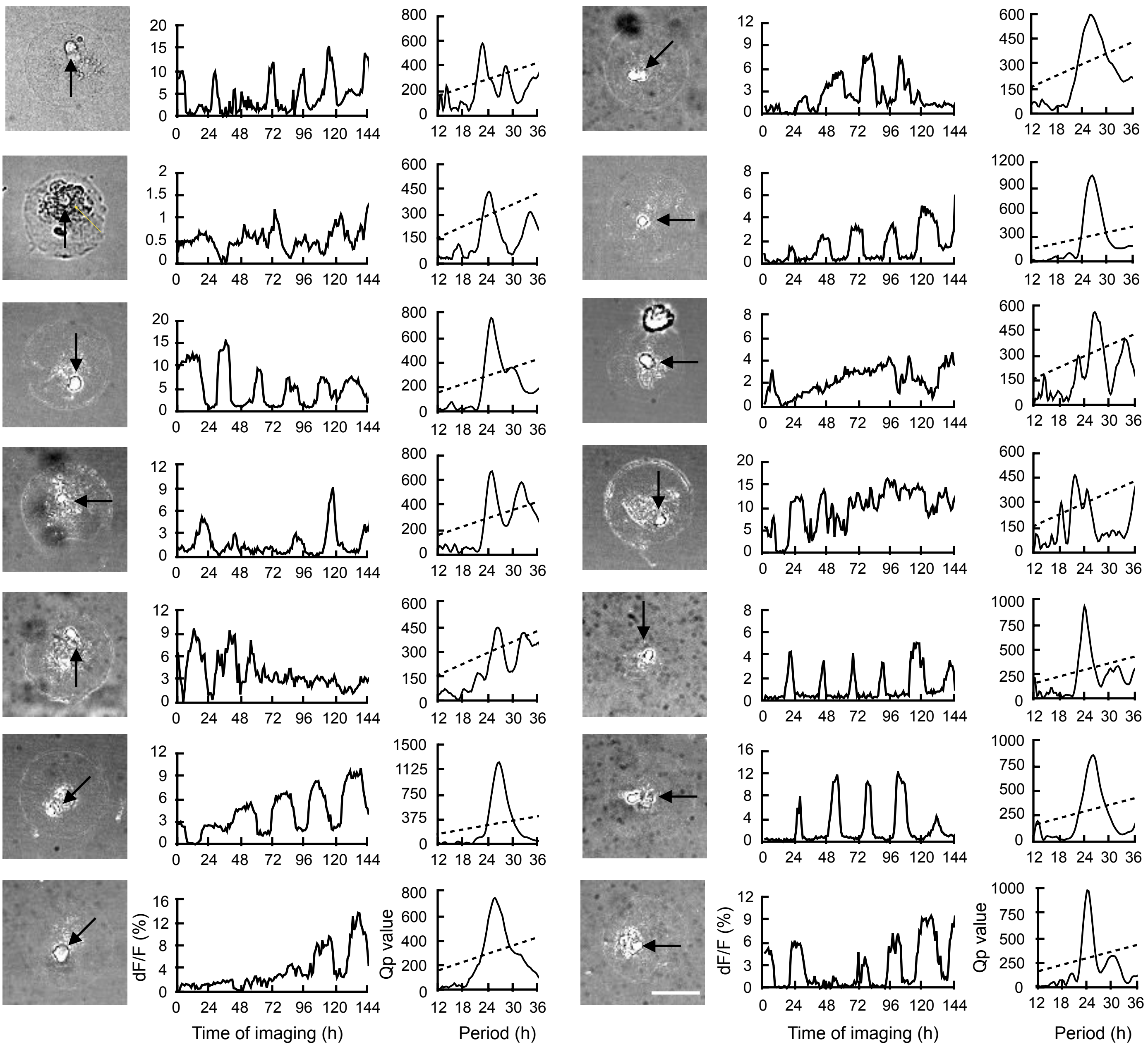

Supplementary Figure 8

Supplementary Figure 8. Circadian Ca^{2+} rhythms in solitary SCN neurons with co-habitant glial cells.

Circadian Ca^{2+} rhythm (left) and Chi-square periodogram (right) of solitary SCN neurons other than the results illustrated in Fig.6. Bright-field photomicrograph (left), time courses of fluorescent signals (middle) and Chi-square periodogram (right) are illustrated.

Neuron with glia (arrhythmic)

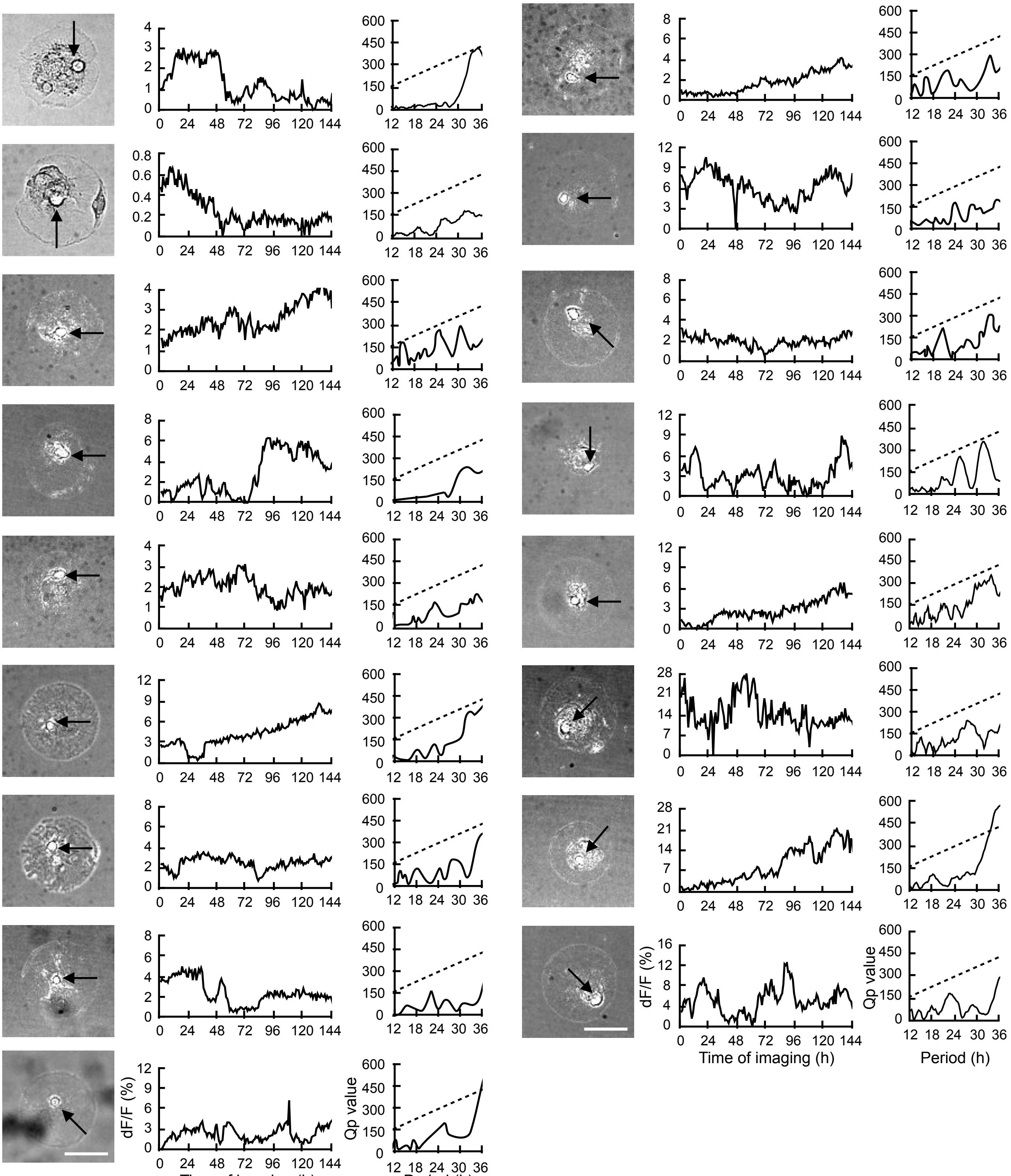

Supplementary Figure 9. Arrhythmic Ca^{2+} expression in solitary SCN neurons with co-habitant glial cells.

Out of 48 islands, 17 examples of arrhythmic Ca^{2+} expression in solitary SCN neurons with co-residence of glial cells. Bright-field photomicrograph (left), the time courses of fluorescent signals (middle) and Chi-square periodogram (right) are illustrated.
